# Diffuse large B‐cell lymphoma presenting as postmenopausal bleeding

**DOI:** 10.1002/ccr3.8173

**Published:** 2023-11-12

**Authors:** Jennifer Cai, Taylor Parnall, Sarah Tomassetti, Xin Qing

**Affiliations:** ^1^ Department of Pathology Harbor‐UCLA Medical Center Torrance California USA; ^2^ University of California Irvine California USA; ^3^ Department of Radiology Harbor‐UCLA Medical Center Torrance California USA; ^4^ Division of Hematology and Oncology Harbor‐UCLA Medical Center Torrance California USA; ^5^ The David Geffen School of Medicine at UCLA Los Angeles California USA

**Keywords:** abnormal uterine bleeding, chemotherapy, diffuse large B‐cell lymphoma, endometrial thickening, primary endometrial lymphoma

## Abstract

**Key Clinical Message:**

Endometrial lymphoma is a rare etiology of abnormal uterine bleeding and endometrial thickening, and often shares similar clinical and imaging characteristics with other uterine malignancies such as endometrial carcinoma. Chemotherapy appears adequate for treating primary endometrial lymphoma.

**Abstract:**

We report a case of primary endometrial diffuse large B‐cell lymphoma to increase awareness of this condition which is essential for correct diagnosis and treatment.

## Graphical abstract

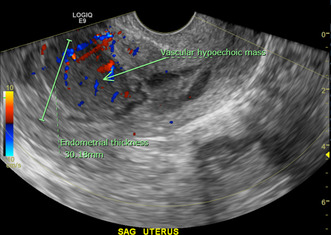

## 1 CASE

**FIGURE 1:**
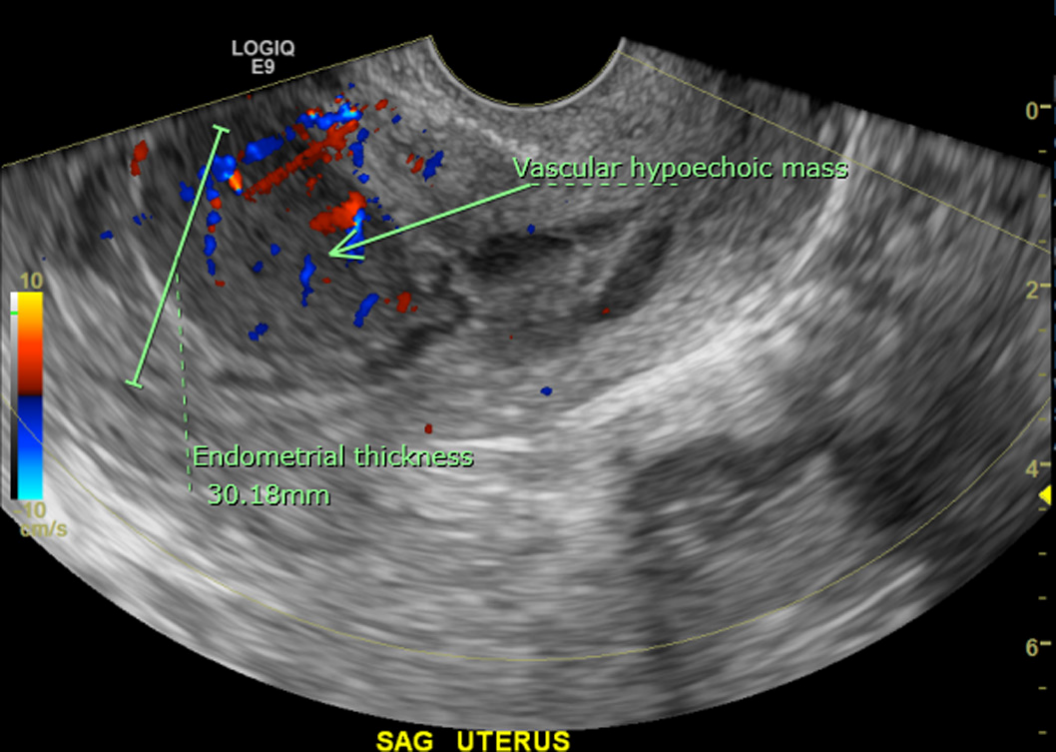
Transvaginal gray‐scale ultrasound image of the uterus in the sagittal plane. The endometrial stripe is markedly thickened to 30 mm with an irregular contour delineated by a small amount of fluid within the endometrial cavity. There is a 3.2 × 2.2 × 3.3 cm^3^ ovoid homogeneous hypoechoic mass within the anterior endometrium with internal color Doppler flow and a low resistance arterial waveform.

**FIGURE 2:**
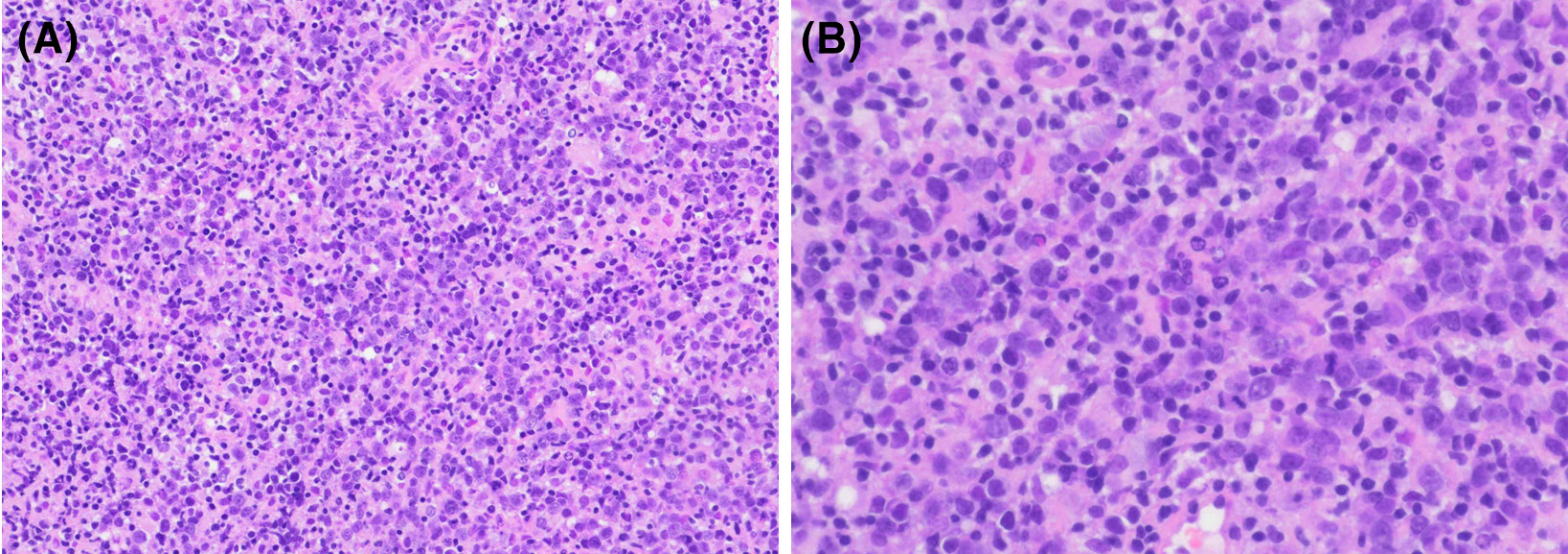
Photomicrograph of the endometrial biopsy. Sheets of large lymphoma cells are present, with atrophic background endometrium. [H&E stain, original magnification, ×200 (A), ×400 (B)].

**FIGURE 3:**
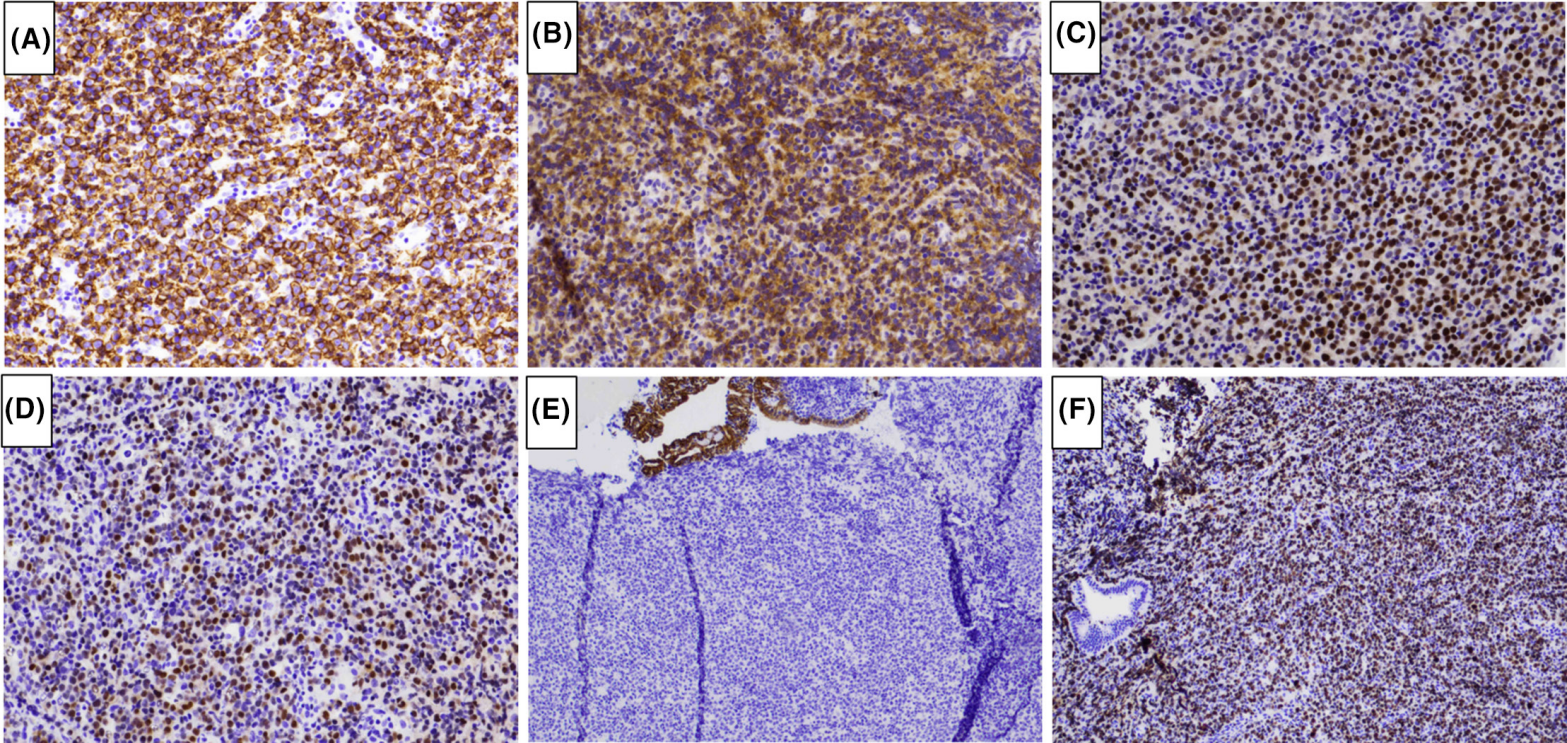
Immunohistochemical characterization of the lymphoma. The lymphoma cells are positive for CD20 (A), CD10 (B), BCL6 (C), and MUM1 (D), and negative for cytokeratin CAM5.2 (E). Ki67 stain showed a high proliferation rate (about 70% of lymphoma cells in cycle) (F). [Immunoperoxidase staining; original magnification, ×400 (A–D), ×100 (E, F)].

A 57‐year old Gravida 3, Para 3 postmenopausal woman with a medical history of hypertension, hyperlipidemia, morbid obesity, and osteoarthritis presented with vaginal bleeding of 10 days duration, requiring 2–5 pads per day. She denied fever, night sweats, or weight loss. Her last menstrual period was 5 years earlier. Her Pap smear was performed 1‐year earlier and was negative for intraepithelial lesion or malignancy with negative HPV. Transvaginal ultrasound revealed markedly irregular heterogeneous mass‐like thickening of the endometrium to 30 mm with internal color Doppler flow (Figure 1), concerning for endometrial carcinoma. An endometrial biopsy demonstrated unexpected diffuse large B‐cell lymphoma (Figure 2). By immunohistochemistry (Figure 3), the lymphoma cells were positive for CD10, CD20, BCL6, and MUM1, and negative for CD3, CD5, BCL2, cyclin D1, TdT, and cytokeratin CAM5.2. Ki67 stain showed a high proliferation rate (about 70%). EBER in situ hybridization was negative. Fluorescence in situ hybridization studies showed negative results for MYC, BCL6, and IGH/BCL2 rearrangements, but with gain/amplification of MYC and BCL6. The findings support the diagnosis of germinal center B‐cell‐like (GCB) subtype of diffuse large B‐cell lymphoma, not otherwise specified (DLBCL, NOS), according to the fifth (2022) edition of the World Health Organization Classification.^1^ Positron emission tomography–computed tomography (PET/CT) scan showed focal avid FDG uptake within the endometrium with an SUV max of 8.95, without other FDG‐avid foci. Bone marrow biopsy showed normocellular (50%) bone marrow with no evidence of lymphoma. The disease was staged as 1E (one extranodal site) according to the Ann Arbor staging system. The patient underwent four cycles of chemotherapy with R‐CHOP (Rituximab, Cyclophosphamide, Hydroxydaunorubicin, Oncovin, and Prednisone). A follow‐up PET/CT scan 6 months after treatment was negative. A pelvis, transabdominal and transvaginal ultrasound 11 months post therapy was normal with the endometrial thickness of 6 mm. As of 12 months post therapy, the patient remains asymptomatic and without signs of recurrence.

## 2 DISCUSSION

Uterine and cervical lymphomas account for only 1.5% of extranodal non‐Hodgkin lymphomas and fewer than 0.5% of all gynecological malignancies.^2^ Most of these lymphomas are secondary involvement by systemic lymphomas.^3^ Primary endometrial lymphomas are extremely rare, and often share similar clinical and imaging characteristics with other uterine malignancies such as endometrial carcinoma.^2^, ^4^ The rarity of the disease and the lack of specific clinical and imaging features may lead to misdiagnosis and inappropriate/suboptimal treatment. Due to its rarity, a standard therapy has not been developed specifically for primary endometrial lymphomas. The treatment options may include surgery, radiation therapy, and chemotherapy, used either alone or in combination. We report this case to emphasize that the differential diagnosis of abnormal uterine bleeding and/or abnormal endometrial thickening should include primary endometrial lymphoma, and that nonsurgical‐based treatment, that is, chemotherapy, appears adequate for treating primary endometrial lymphoma.

## AUTHOR CONTRIBUTIONS

**Jennifer Cai:** Conceptualization; investigation; methodology; project administration; visualization; writing – original draft; writing – review and editing. **Taylor Parnall:** Methodology; resources; visualization; writing – review and editing. **Sarah Tomassetti:** Investigation; project administration; resources; writing – review and editing. **Xin Qing:** Conceptualization; data curation; supervision; writing – review and editing.

## FUNDING INFORMATION

None.

## CONFLICT OF INTEREST STATEMENT

None.

## ETHICS STATEMENT

Ethical review and approval of the study are not applicable in this case.

## CONSENT

Written informed consent was obtained from the patient to publish this report in accordance with the journal's patient consent policy.

## Data Availability

No datasets were generated or analyzed during the current study.
